# ^23^Na MRI: inter-reader reproducibility of normal fibroglandular sodium concentration measurements at 3 T

**DOI:** 10.1186/s41747-024-00465-x

**Published:** 2024-06-10

**Authors:** Otso Arponen, Mary A. McLean, Muzna Nanaa, Roido Manavaki, Gabrielle C. Baxter, Andrew B. Gill, Frank Riemer, Aneurin J. Kennerley, Ramona Woitek, Joshua D. Kaggie, William J. Brackenbury, Fiona J. Gilbert

**Affiliations:** 1 Department of Radiology, School of Clinical Medicine, University of Cambridge, Cambridge Biomedical Campus, Box 218, Cambridge, CB2 0QQ UK; 2 Department of Radiology, Mohn Medical Imaging and Visualization Centre (MMIV), Haukeland University Hospital, Bergen, Norway; 3 York Biomedical Research Institute, University of York, York, UK; 4 Department of Sports and Exercise Science, Institute of Sport, Manchester Metropolitan University, Manchester, UK; 5 Research Center for Medical Image Analysis and AI (MIAAI), Danube Private University, Krems, Austria; 6 Department of Biology, University of York, York, UK

**Keywords:** Breast neoplasms, Healthy volunteers, Magnetic resonance imaging, Reproducibility of results, Sodium

## Abstract

**Background:**

To study the reproducibility of ^23^Na magnetic resonance imaging (MRI) measurements from breast tissue in healthy volunteers.

**Methods:**

Using a dual-tuned bilateral ^23^Na/^1^H breast coil at 3-T MRI, high-resolution ^23^Na MRI three-dimensional cones sequences were used to quantify total sodium concentration (TSC) and fluid-attenuated sodium concentration (FASC). B_1_-corrected TSC and FASC maps were created. Two readers manually measured mean, minimum and maximum TSC and mean FASC values using two sampling methods: large regions of interest (LROIs) and small regions of interest (SROIs) encompassing fibroglandular tissue (FGT) and the highest signal area at the level of the nipple, respectively. The reproducibility of the measurements and correlations between density, age and FGT apparent diffusion coefficient (ADC) values were evaluatedss.

**Results:**

Nine healthy volunteers were included. The inter-reader reproducibility of TSC and FASC using SROIs and LROIs was excellent (intraclass coefficient range 0.945−0.979, *p* < 0.001), except for the minimum TSC LROI measurements (*p* = 0.369). The mean/minimum LROI TSC and mean LROI FASC values were lower than the respective SROI values (*p* < 0.001); the maximum LROI TSC values were higher than the SROI TSC values (*p* = 0.009). TSC correlated inversely with age but not with FGT ADCs. The mean and maximum FGT TSC and FASC values were higher in dense breasts in comparison to non-dense breasts (*p* < 0.020).

**Conclusions:**

The chosen sampling method and the selected descriptive value affect the measured TSC and FASC values, although the inter-reader reproducibility of the measurements is in general excellent.

**Relevance statement:**

^23^Na MRI at 3 T allows the quantification of TSC and FASC sodium concentrations. The sodium measurements should be obtained consistently in a uniform manner.

**Key points:**

• ^23^Na MRI allows the quantification of total and fluid-attenuated sodium concentrations (TSC/FASC).

• Sampling method (large/small region of interest) affects the TSC and FASC values.

• Dense breasts have higher TSC and FASC values than non-dense breasts.

• The inter-reader reproducibility of TSC and FASC measurements was, in general, excellent.

• The results suggest the importance of stratifying the sodium measurements protocol.

**Graphical Abstract:**

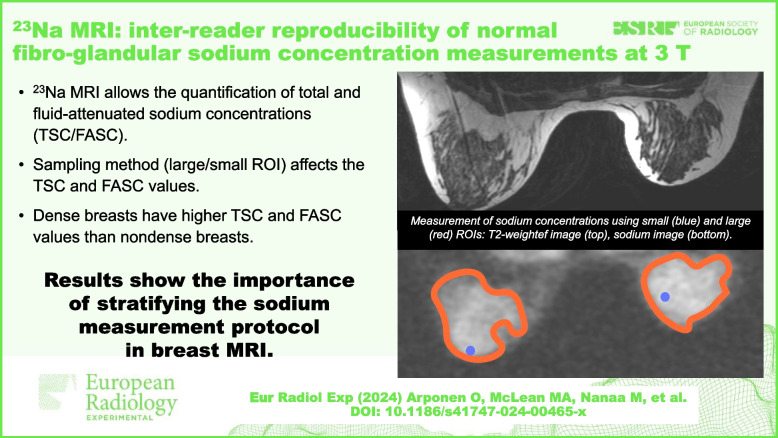

**Supplementary Information:**

The online version contains supplementary material available at 10.1186/s41747-024-00465-x.

## Background

Imaging methods to better differentiate malignant and benign breast lesions and to evaluate treatment response in breast cancer are of great clinical interest. Sodium magnetic resonance imaging (MRI) based on ^23^Na nuclei allows the measurement of total sodium concentration (TSC) [1, 2], a combination of intra- and extracellular sodium concentrations. Physiological extracellular sodium concentration ranges from 135 to 155 mmol/L and is usually ten times higher compared to the intracellular concentration of 5–15 mmol/L [1, 3]. Although ^23^Na MRI has not been translated to routine clinical practise, preclinical evidence from a murine breast model [4] and clinical studies [5, 6] with ^23^Na MRI have shown that the TSC is elevated in malignant breast tumours compared to corresponding healthy tissues [5, 6] or benign breast lesions [6]. Furthermore, chemotherapy has been shown to reduce tumoural TSC in a preclinical breast cancer model [4] and in patients [5–8]. ^23^Na MRI has not been able to differentiate between intra- and extracellular sodium concentrations in human studies [9]. However, studies have shown that fluid-attenuated sodium concentration (FASC), a mathematical approximation emphasising the concentration of sodium in the intracellular compartment, is elevated in tumours (*e.g.,* in gliomas [10] and high-grade serous ovarian cancer [1]) in comparison to normal appearing tissues.

^23^Na MRI is limited by low ^23^Na concentrations in biological tissues, rapid bi-exponential signal decays, and a low gyromagnetic ratio of ^23^Na that requires high gradient magnetic field slew rates [11, 12]. Early ^23^Na MRI studies lacked clinical commercial coils, thus requiring the development of new coil designs [12]. Early studies also used 1.5 T magnetic field strengths, which resulted in lower signal-to-noise rations that have been achieved in more recent studies at 3 and 7 T [5, 6]. ^23^Na MRI requires long scanning times, which results in low spatial resolutions. Zaric et al. reported scanning times of 16–20 min and resolutions of 1.8 × 1.8 × 5 mm^3^ [6] and 3 × 3 × 3 mm^3^ [8] at 7 T suggesting that the method is potentially translatable to clinical work.

A key aspect towards clinical translation of novel quantitative imaging methods is ensuring method robustness through the assessment of inter-reader reproducibility. Zaric et al. [6] reported that there were no statistical differences in mean TSC measurements acquired with circular region of interest (ROI) of no fixed size that were placed on healthy fibroglandular tissue (FGT) by two readers. Furthermore, they later reported excellent reproducibility of TSC reduction after 1 and 2 cycles of neoadjuvant chemotherapy with ROIs covering the full cross-sectional area of the tumour on at least three different sections [8]. Indeed, although these studies suggest excellent reproducibility of TSC values, the effect of ROI sampling methods and the chosen descriptive value on reproducibility in ^23^Na MRI remains unknown.

This work aims to study whether the quantitative values (mean, minimum, and maximum) of TSC and FASC are reproducible between two readers using targeted large regions of interest (SROIs) and small regions of interest (SROIs) in healthy volunteers. We hypothesise that this information could help to standardise the reporting, and eventually potential clinical translation, of ^23^Na MRI.

## Methods

### Study volunteers

The National Research Ethics Committee approved this study (08-H0311-117). Following written informed consent, ten healthy volunteers without known breast conditions were recruited and imaged in 2019 and 2020.

### MRI protocol

Females were scanned in a prone position with a dual-tuned bilateral ^23^Na/^1^H breast coil (Rapid Biomedical, Rimpar, Germany) on a 3-T system (MR750, GE Healthcare, Waukesha, WI, USA). The coil consisted of four sodium transmit/receive channels, two per breast, and sixteen receive-only proton channels. Two agar phantoms with known sodium concentrations (40 mmol/L and 80 mmol/L) were placed within the coil to scale the high-resolution and B_1_-maps to quantify the TSC and FASC values. A high-resolution ^23^Na MRI three-dimensional cones ultra-short echo time trajectory sequence was used to acquire TSC data for quantification [13]. The following parameters were used: repetition time/echo time 100/0.46 ms; flip angle 90°; voxel size 3 × 3 × 6 mm^3^; field of view 36 × 36 × 36 cm^3^; averages 4; interleaves 1,402; scan time 9:21 min:s. A second sequence with a matching three-dimensional cones trajectory was acquired, with an additional inversion recovery pulse for FASC value evaluation [1]. This scan was acquired with inversion time 30 ms; repetition time/echo time 250/0.46 ms; averages 2; interleaves 1,402, scan time 11:41 min:s; and a field of view of either 36 × 36 × 36 cm^3^ or 40 × 40 × 40 cm^3^. Low-resolution sodium images were acquired at flip angles of 40° and 80° for double-angle method B_1_ mapping (repetition time/echo time 100/0.46 ms; interpolated voxel size 3 × 3 × 3 mm^3^; true resolution 6.75 mm; field of view 36 × 36 × 36 cm^3^; averages 4; interleaves 197; scan time 1:19 min:s per flip angle) [14]. Proton-only images were acquired using a three-dimensional T1-weighted fast spoiled gradient echo sequence (repetition time/echo time 5.3/2.1 ms; voxel size 1.4 × 1.4 × 2.8 mm^3^; field of view 35 × 35 × 15.7 cm^2^; scan time 41 s). Eight volunteers underwent diffusion-weighted imaging performed using a single-shot echo-planar imaging sequence with repetition time/echo time 4,000/94.2 ms; voxel size 2.8 × 2.8 × 4 mm^3^; field of view 36 × 36 × 16 cm^3^; acceleration factor 2; averages 4; *b*-values 0, 100, 500, 1,000, 1,500, 2,000 and 2,500 s/mm^2^ (total scan time 10:16 min:s).

### Postprocessing of the sodium images

To avoid bias introduced by the scanner default, which scales the Digital imaging and Communications in Medicine (DICOM) images to the maximum, analysis of the sodium data was performed on raw MATLAB (version 2019a; MathWorks, Natick, MA, USA) data rewritten as DICOM images with common scaling between series. Postprocessing analysis also enabled the assessment of TSC and FASC values from quantitative maps. The TSC and FASC maps were corrected using ^23^Na B_1_ maps, which used the double-angle method for B_1_ mapping [15]. DICOM images of the B_1_ maps were also output of this relative or fractional B_1_ × 1,000 for inspection of ^23^Na B_1_ effects.

**Fig. 1:**
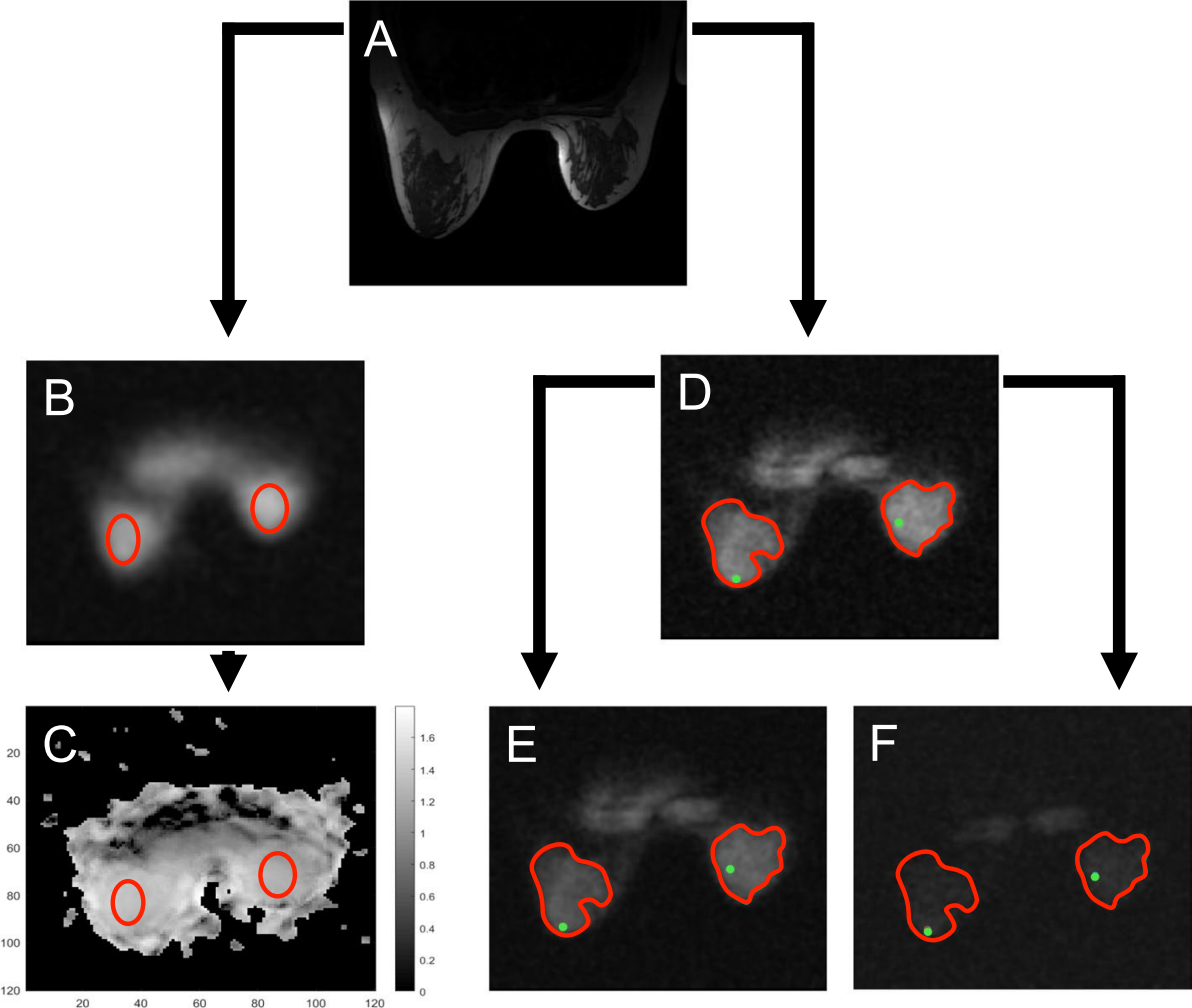
The measurement of total and fluid-attenuated sodium concentration in fibroglandular tissue (FGT). FGT at the nipple level was first identified on T1- and T2-weighted images (T2-weighted image shown in **a**). The readers placed as large ellipsoid region of interest (ROIs) (red) encompassing only FGT (**b**) as possible on the low-resolution 80° B_1_ image; ROIs were then propagated on the B_1_ image (**c**). The readers delineated the confidently visualisable FGT (SROI, red) and placed a circular small region of interest on the FGT with the highest signal intensity (SROI, green) (**d**). These ROIs were then propagated in the scaled high-resolution (**e**) and inversion recovery (**f**) images. The boundaries of the ROIs were thickened for illustrative purposes

Visual inspection of the B_1_ maps (Fig. 1) suggested that B_1_ levels varied relatively little within breasts despite the differences observed between the location of the phantoms and between the left and right breasts. Since the methods developed here were intended to be applied also in a patient study where B_1_ maps were not acquired for more rapid imaging, analysis was simplified to a single scale factor per breast to account for different relative B_1_ values in the phantom.

Two readers (radiology resident (O.A.), Reader 1; radiologist (M.N.), Reader 2, with 7 years of experience in breast imaging) performed the analyses by drawing two-dimensional ROIs using OsiriX DICOM Viewer (v.11.0.4., Pixmeo SARL, Bernex, Switzerland). First, the radiologists independently determined the nipple level using the T1- and T2-weighted images. The radiologists then placed large bilateral ellipsoid ROIs encompassing only FGT on the low-resolution images that were acquired with a flip angle of 80°. These ROIs were as large as possible and placed at the nipple level. Subsequently, they placed ellipsoid ROIs covering 80% of the visible phantom. These ROIs were propagated on the B_1_ map to acquire B_1_ correction values for the FGT and phantom.

For the assessment of TSC and FASC, the radiologists drew independently freehand circular LROIs covering as large areas of the FGT as possible, and circular SROIs of approximately 5 mm in diameter on the area with the highest signal intensity on the high-resolution images at the nipple level. To ensure that the regions consisted of FGT, the radiologists referred to the proton images while drawing the ROIs. Subsequently, they placed ellipsoid ROIs covering 80% of the visible phantom on the image that contained the largest part of the phantom that had the higher signal intensity. Circular LROIs were also drawn on the high-resolution sodium image outside of the breast to quantify noise. The four types of ROIs (LROI, SROI and the ROIs within the phantom and outside of the breast to quantify noise) were transposed onto the scaled sodium high-resolution and inversion recovery images to produce quantitative mean, minimum and maximum signal intensities that were converted to total and fluid-attenuated sodium concentrations (Fig. 1).

### TSC and FASC quantification

Following the method proposed by Deen et al. [1], non-B_1_-inhomogeneity corrected TSCs (TSCcrude) were calculated for the mean, minimum and maximum LROI and SROI signal intensity values according to Eq. 1; B_1_-inhomogeneity corrected TSCs (TSCcorrected) were acquired using Eqs. 2 and 3.

1 $$\textit{TSCcrude}=Cphantom\times\frac{SItissue}{SIphantom}$$

2 $$\textit{aFA}\mathit=\textit{nFA}\mathit\times\textit{B}_1$$

3 $$\textit{TSCcorrected}\mathit=Cphantom\times\frac{SItissue}{SIphantom}\mathit\times\frac{\textit{sin}(aFAphantom)}{sin(aFAtissue)}\mathit\times\frac{{B}_1phantom}{{B}_1tissue}$$

Where TSC refers to the total sodium concentration acquired with the ROI in question (LROI or SROI), Cphantom to the sodium concentration of the phantom (80 mmol/L), SI to the signal intensities of the fibroglandular tissue and phantom, nFA to the nominal flip angle used when capturing the high-resolution images (90°), B_1_ to the B_1_ values of the fibroglandular tissue and phantom, and aFA to the B_1_-corrected actual flip angle calculated separately for the fibroglandular tissue and phantom. The sine terms correct for transmit B_1_ sensitivity and the final B_1_ correction is for the received B_1_ inhomogeneity.

Following [1], the mean inversion recovery signal intensities were converted into FASC values, while taking noise and the differing acquisition parameters into account, as follows:

4 $$\textit{FASC}\mathit=TSCcorrected\mathit\times\frac{FOVVhighres}{FOVVIR}\mathit\times\sqrt{\frac{NEXhighres}{NEXIR}}\mathit\times\frac{SNRIR}{SNRhighres}$$

Where FASC and TSC refer to the fluid-attenuated and total sodium concentrations acquired with the ROI in question (LROI or SROI). The FOVV terms correct for differences in the high-resolution (FOVVhighres) and inversion recovery (FOVVIR) field of view volumes. NEX refers to the number of averaged exams; the NEX terms correct for any difference in the averages between the high-resolution (NEXhighres) and inversion recovery (NEXIR) images. SNRhighres and SNRIR refer to the fibroglandular tissue signal-to-noise ratios of the high-resolution and inversion recovery images, respectively, and were determined as follows:

5 $$\textit{SNRIR}\mathit={\left(\mathit{IRSItissure}-{IRISInoise}\right)}/\mathit{IRSInoiseSD}$$

6 $$\textit{SNRhighres}\mathit={\left(\mathit{SItissure}-{SInoise}\right)}/\mathit{SInoiseSD}$$

where IRSItissue and IRSInoise stand for the mean inversion recovery signal intensities of FGT and noise and SInoiseSD refers to the standard deviation of noise signal intensity determined from the high-resolution image. SItissue and SInoise stand for the mean high-resolution signal intensities of FGT and noise; IRSInoiseSD refers to the standard deviation of noise signal intensity determined from the inversion recovery image.

### Diffusion-weighted imaging measurements and density assessment

The two readers placed as large ROIs as possible on the apparent diffusion coefficient (ADC) maps in agreement to quantify the FGT ADC values. The readers visually evaluated the anatomical series together and classified the breasts as non-dense or dense (≤ 50% and > 50% of the breast composed of FGT, respectively).

### Statistical analysis

Data were analysed using SPSS (Mac version 29, 1989–2023 SPSS Inc., Chicago, USA). Unless otherwise stated, continuous variables are presented as means ± standard deviations (SDs). Statistical significance was set at *p* < 0.05. Mean, minimum and maximum LROI and SROI TSC and mean FASC values for left and right breasts were independently used for analyses (*i.e.*, analyses were conducted on a breast level). Furthermore, unless otherwise noted, when analysing the B_1_-corrected values, the effect of differences in phantom measurements was minimised by using the phantom signal intensity and B_1_ measurements of Reader 1.

Normality of the data was assessed with the Kolmogorov–Smirnov test, and the paired-sample *t*-test (normal distribution) and Wilcoxon signed-rank test (non-normal distribution) were employed to compare the statistical difference between the mean, minimum and maximum TSC and FASC values acquired using the two ROI sampling methods and between the two breasts (right *versus* left). The intraclass correlation coefficient test was used to evaluate the linear average inter-reader correlations of the two readers’ TSC and FASC mean, minimum and maximum values. The independent samples *t*-test was used to test the association between the TSC and FASC measurements and breast density (non-dense *versus* dense) and the equality of variances was confirmed with Levene’s test when the distribution was normal; in case of non-normal distribution, the Mann–Whitney *U* test was used. As the ages of the participants and the fibroglandular tissue ADC values and were not normally distributed in the cohort according to the Kolmogorov–Smirnov test, we used the Spearman correlation test to evaluate the correlation between the TSC and FASC values, ADC values and age.

## Results

**Fig. 2:**
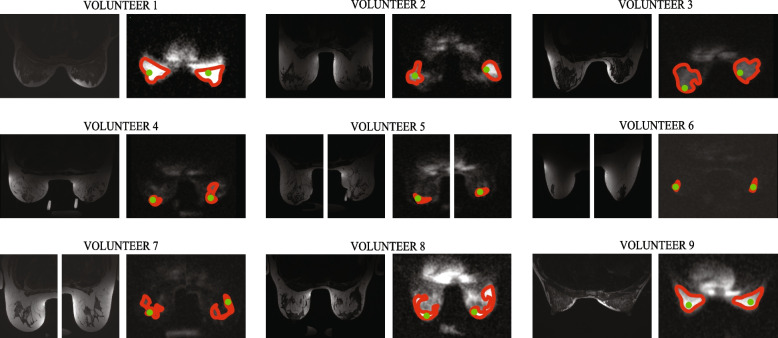
An illustrative figure of large (red) and small (green) regions of interest. The boundaries of the ROIs were thickened for illustrative purposes

Ten healthy female volunteers were prospectively recruited. One volunteer was excluded from the analyses as the phantom was poorly visible preventing reliable quantitative sodium measurements; therefore, the final sample consisted of 9 volunteers (Fig. 2). The mean age of the included volunteers was 38.2 ± 15.6 years (mean ± standard deviation), range 24–66 years. Five of the nine volunteers (55.6%) had non-dense breasts (≤ 50% FGT).

Phantom segmentation had a significant effect on the TSC and FASC FGT values (Supplementary Table S1). The B_1_-corrected mean, minimum and maximum TSC values were approximately 40% lower than the non-B_1_-corrected concentrations (*p* < 0.001, Supplementary Table S2). There were no statistically significant differences between right and left breasts with or without B_1_ corrections (Table 1). The results reported hereafter were obtained using phantom-stratified and B_1_-corrected values.

**Table 1:** Mean total sodium concentrations of healthy volunteers’ fibroglandular tissue of the right and left breasts without and with B_1_ corrections

		Non-B_1_-corrected (crude)			B_1_-corrected^a^		
ROI method	Reader	Right (mmol/L)	Left (mmol/L)	*p*-value	Right (mmol/L)	Left (mmol/L)	*p*-value
Large ROI mean	Reader 1^a^	101.0 ± 37.4	92.0 ± 23.2	0.15	57.7 ± 19.7	53.6 ± 13.0	0.34
	Reader 2^a^	109.9 ± 39.5	100.9 ± 28.6	0.23	62.8 ± 20.1	58.1 ± 14.5	0.29
Small ROI mean	Reader 1^a^	137.5 ± 54.0	126.9 ± 35.6	0.25	77.5 ± 26.4	73.4 ± 17.8	0.52
	Reader 2^a^	137.8 ± 49.8	128.7 ± 30.5	0.41	78.5 ± 25.1	74.4 ± 16.1	0.56

The results are shown for the readers’ large and small regions of interest (ROIs); *p*-value denotes the statistical difference between the right- and left-sided measurements

^a^Phantom’s signal intensity and B_1_ measurement measured by Reader 1 was used to minimise the effect of differences caused by phantom ROI placement that contributes to B_1_-corrected measurements

The mean and minimum LROI TSCs (55.7 ± 16.3/60.4 ± 17.2 mmol/L and 20.4 ± 6.1/30.3 ± 8.5 mmol/L for Reader 1/Reader 2, respectively) were lower than the mean and minimum SROI TSCs (75.4 ± 21.9/76.5 ± 20.6 mmol/L and 68.4 ± 22.2/70.9 ± 20.2 mmol/L, respectively) (*p* < 0.001 for all; Table 2). Although differences between the maximum LROI TSCs (82.5 ± 22.7/82.4 ± 22.4 mmol/L) were similar to the maximum SROI TSCs (81.5 ± 22.1/81.0 ± 21.3 mmol/L), the maximum LROI TSC values were statistically higher than the maximum SROI TSC values (*p* = 0.009/0.017). The mean FASC values acquired using the LROI were significantly lower than those acquired with the SROI (17.2 ± 6.6/18.9 ± 7.3 mmol/L *versus* 25.2 ± 10.7/25.1 ± 10.2 mmol/L, *p* < 0.001 for both; Table 2).

**Table 2:** Mean, minimum and maximum total sodium concentrations and mean fluid-attenuated sodium concentrations of healthy volunteers’ fibroglandular tissue areas

	Mean TSC			Minimum TSC			Maximum TSC			Mean FASC		
	Large ROI (mmol/L)	Small ROI (mmol/L)	*p*-value	Large ROI (mmol/L)	Small ROI (mmol/L)	*p*-value	Large ROI (mmol/L)	Small ROI (mmol/L)	*p*-value	Large ROI (mmol/L)	Small ROI (mmol/L)	*p*-value
Reader 1^a^	55.7 ± 16.3	75.4 ± 21.9	< 0.001	20.4 ± 6.1	68.4 ± 22.2	< 0.001	82.5 ± 22.7	81.5 ± 22.1	0.009	17.2 ± 6.6	25.2 ± 10.7	< 0.001
Reader 2^a^	60.4 ± 17.2	76.5 ± 20.6	< 0.001	30.3 ± 8.5	70.9 ± 20.2	< 0.001	82.4 ± 22.4	81.0 ± 21.3	0.017	18.9 ± 7.3	25.1 ± 10.2	< 0.001

*FASC* Fluid-attenuated sodium concentration, *ROI* Region of interest, *TSC* Total sodium concentration

The results are shown for the readers’ large and small regions of interest (ROIs)

^a^Phantom’s signal intensity and B_1_ measurement measured by Reader 1 was used to minimise the effect of differences caused by phantom ROI placement that contributes to B_1_-corrected measurements

The inter-reader reproducibility of the mean, minimum and maximum LROI TSCs were 0.962 (95% confidence interval (CI) 0.657-0.990, *p* < 0.001), 0.086 (95% CI -0.404−0.527 (*p* = 0.369)) and 0.979 (95% CI 0.943−0.992, *p* < 0.001). The inter-reader reproducibility of the SROI mean, minimum and maximum TSCs were 0.973 (95% CI 0.928−0.990, *p* < 0.001), 0.956 (95% CI 0.886−0.98, *p* < 0.001) and 0.974 (95% CI 0.932−0.990 (*p* < 0.001)), respectively. The inter-reader reproducibility of the mean FASC values were 0.949 (95% CI 0.812−0.983, *p* < 0.001) and 0.945 (95% CI 0.852−0.979, *p* < 0.001) using LROIs and SROIs, respectively. The results are shown in Table 3.

**Table 3:** Mean, minimum and maximum B_1_-corrected total sodium concentrations and mean fluid-attenuated sodium concentrations of healthy volunteers’ fibroglandular tissue areas

		Mean TSC		Minimum TSC		Maximum TSC		Mean FASC	
ROI method	Reader	Concentration (mmol/L)	ICC (95% CI)	Concentration (mmol/L)	ICC (95% CI)	Concentration (mmol/L)	ICC (95% CI)	Concentration (mmol/L)	ICC (95% CI)
Large ROI	Reader 1^a^	55.7 ± 16.3	0.962 (0.657−0.990)*	20.4 ± 6.1	0.086 (-0.404−0.527)**	82.5 ± 22.7	0.979 (0.943−0.992)*	17.2 ± 6.6	0.949 (0.812−0.983)*
	Reader 2^a^	60.4 ± 17.2		30.3 ± 8.5		82.4 ± 22.4		18.9 ± 7.3	
Small ROI	Reader 1^a^	75.4 ± 21.9	0.973 (0.928−0.990)*	68.4 ± 22.2	0.956 (0.886−0.984)*	81.5 ± 22.1	0.974 (0.932−0.990)*	25.2 ± 10.7	0.945 (0.852−0.979)*
	Reader 2^a^	76.5 ± 20.6		70.9 ± 20.2		81.0 ± 21.3		25.1 ± 10.2	

*CI* Confidence interval, *FASC* Fluid-attenuated sodium concentration, *ICC* Intraclass correlation coefficient, *ROI* Region of interest, *TSC* Total sodium concentration

The results are shown for the readers’ large and small ROIs. The inter-reader repeatability was tested using the intraclass correlation coefficients

^a^The phantom’s signal intensity and B_1_ measurement measured by Reader 1 was used to minimise the effect of differences caused by phantom ROI placement that contributes to B_1_-corrected measurements. * Statistically significant (*p* < 0.001) ** Statistically insignificant (*p* = 0.369)

The FGT TSCs and FASCs were lower in non-dense breasts in comparison to dense breasts; only minimum total sodium concentrations acquired using LROIs by the two readers and mean FASC, as measured by Reader 1 with SROI, were not significantly different between the non-dense and dense breasts (*p* ≥ 0.067) (Table 4). Mean SROI TSC (-0.645, *p* = 0.004) for Reader 1 and mean LROI and SROI TSCs (LROI, *r* = -0.527 (*p* = 0.025), SROI, *r* = **-**0.546 *p* = 0.019) for Reader 2, minimum SROI TSC (*r* = -0.674, *p* = 0.002 / *r* = -0.548, *p* = 0.019 (Reader 1/Reader 2)) and maximum LROI TSC (*r* = -0.527, *p* = 0.025 / *r* = -0.531, *p* = 0.023 (Reader 1/Reader 2)) and SROI TSCs (*r* = -0.536, *p* = 0.022) for Reader 1 were inversely correlated with age. Apart from LROI FASC, concentration as measured by Reader 2 (*r* = -0.496, *p* = 0.036)), FASC values did not statistically correlate with age. ADC values (1180.8 ± 121.0 mm^2^/s, mean ± SD) could be measured for 12 breasts. ADC values did not correlate with TSC or FASC values.

**Table 4:** Sodium measurements from nine healthy participants using large and small ROIs and their association with breast density groups (≤ 50% (*n* = 10 breasts) and > 50% (*n* = 8 breasts))

	Reader 1^a^			Reader 2^a^		
	Non-dense breasts	Dense breasts	*p*-value	Non-dense breasts	Dense breasts	*p*-value
Large ROI measurements						
Mean TSC (mmol/L)	46.1 ± 12.2	67.6 ± 12.7	0.002	49.8 ± 11.1	73.7 ± 14.0	< 0.001
Min. TSC (mmol/L)	19.3 ± 7.7	21.8 ± 3.3	0.696	29.3 ± 6.5	31.6 ± 10.9	0.574
Max. TSC (mmol/L)	68.2 ± 16.1	100.4 ± 16.3	< 0.001	68.8 ± 16.9	99.4 ± 16.0	0.001
Mean FASC (mmol/L)	14.0 ± 5.8	21.2 ± 5.5	0.017	15.5 ± 5.8	23.2 ± 7.0	0.020
Small ROI measurements						
Mean TSC (mmol/L)	61.1 ± 14.8	93.4 ± 14.7	< 0.001	63.9 ± 15.0	92.2 ± 15.3	0.001
Min. TSC (mmol/L)	54.1 ± 14.5	86.3 ± 16.5	< 0.001	58.0 ± 13.0	86.9 ± 15.6	< 0.001
Max. TSC (mmol/L)	67.5 ± 16.2	98.9 ± 14.9	< 0.001	68.6 ± 16.6	96.4 ± 16.0	0.003
Mean FASC (mmol/L)	21.1 ± 8.8	30.3 ± 11.2	0.067	20.0 ± 8.4	31.5 ± 8.9	0.012

*FASC* Fluid-attenuated sodium concentration, *ROI* Region of interest, *TSC* Total sodium concentration

Data are given as mean ± standard deviation. The *p*-value denotes the statistical difference between non-dense and dense breasts

^a^The phantom’s signal intensity and B_1_ measurement measured by Reader 1 was used to minimise the effect of differences caused by phantom ROI placement that contributes to B_1_-corrected measurements

## Discussion

Our main result is that the inter-reader reproducibility of the mean and maximum total and fluid-attenuated sodium concentrations of fibroglandular tissue between the readers is very good, except for the minimum values for the large region of interests. The poor reproducibility in the minimum sodium concentration values using the LROI is unsurprising as it is likely heavily influenced by the partial volume effect with fat contributing little to sodium signal and noise [15, 16]. With the exception of minimum values LROI TSC, we found that healthy females with dense breasts have higher FGT TSCs in comparison to females with non-dense breasts. Age was inversely correlated with total tissue sodium concentrations.

Although the reproducibility of TSC values has been reported, the effect of ROI sampling methods and the chosen descriptive value (*i.e.*, mean, minimum or maximum) on reproducibility in ^23^Na MRI remains unknown (Table 5). Zaric et al. [6] reported that there were no statistical differences in mean TSC measurements of healthy FGT (ROI size 85 ± 18 mm^2^ (mean ± standard deviation), range 47–123 mm^2^)) between two readers and further reported [8] perfect reproducibility (intraclass correlation coefficients 1.00 (95% CI 0.99−1.00) of TSC reduction after 1 and 2 cycles of neoadjuvant chemotherapy with ROIs covering the full cross-sectional area of the tumour on at least three different sections. Our results support the previous notation of excellent reproducibility. However, the fact that the use of the mean and maximum LROI and mean, minimum and maximum SROI result in statistically significantly different sodium concentrations, the sodium measurements should be performed in a standardised way similar the quantification of ADC value [17, 18].

**Table 5:** A summary of articles focusing on ROI delineation methods for ^23^Na MRI in breasts

First author, year [reference number]	Number of subjects (mean age, years)	Sodium coil / Scanner	ROI sampling method	Mean FGT total sodium concentration	Mean lesion total sodium concentration
Ouwerkerk, 2007 [5]	22 patients; 3 with benign lesions and 19 with malignant lesions	Custom made bilateral phased array coil (NND, Medrad, Indianola, PA, USA) / 1.5 T (NND, General Electric Healthcare)	NR (ROI size 1,000−2,000 pixels)	Patients with cancer: 34 ± 13 mmol/kg	Benign tumours, 26 ± 5 mmol/L; malignant tumours, 53 ± 16 mmol/L
Jacobs, 2010 [7]	18 patients (49 ± 8)	Custom made sodium coil / 1.5 T (NND, General Electric Healthcare)	NR (ROI size: 1,000−2,000 pixels)	NR	Responders (*n* = 15): PreNACT 66 ± 18 mmol/L; PostNACT 48.4 ± 8 mmol/L (within 14 days after the 1st cycle) Non-responders (*n* = 3): PreNACT 52 ± 8 mmol/L; PostNACT 56 ± 2 mmol/L (within 14 days after the 1st cycle)
Jacobs, 2011 [19]	6 patients (43.2 ± 12.5)	Custom made sodium coil / 1.5 T (NND, General Electric Medical Systems)	NR (ROI size: 1,000−2,000 pixels)	NR	PreNACT 55.2 mmol/L; PostNACT 45.0 mmol/L (7-8 days after the 1st cycle)
Zaric, 2016 [6]	5 healthy volunteers (NR^a^) / 17 patients with cancer (56 ± 12)	Dual-tuned sodium and proton-tuned bilateral coil (QED) / 7 T (Magnetom; Siemens Healthineers)	Circular ROIs of no fixed size were placed on the solid parts of tumour while avoiding partial volume effect from fatty parenchyma and excluding cystic necrotic areas. ROIs of same size were defined in similar positions on the contralateral side in FGT	Healthy volunteers: 36 ± 2 mmol/kg (Reader 1) and 37 ± 1 mmol/kg (Reader 2) Patients with cancer: 35 ± 3 mmol/kg	Benign tumours: 47 ± 8 mmol/kg Malignant tumours: 69 ± 10 mmol/kg
Zaric, 2021 [8]	3 healthy volunteers (NR^a^) / 15 patients with cancer (60 ± 10)	Dual-tuned sodium and proton-tuned bilateral coil (QED) / 7 T (Magnetom; Siemens Healthineers)	NR (ROIs covered the full cross-section of the tumour on at least three different sections)	49.0 ± 3.0 mmol/L	PreNACT: 70.6 ± 6.0 mmol/L PostNACT (patients with pCR): 60.9 ± 5.0 mmol/L (after one cycle) 49.1 ± 5.0 mmol/L (after two cycles) PostNACT (patients without pCR): 49.1 ± 5.0 mmol/L (after one cycle) 69.6 ± 9.0 mmol/L (after two cycles)
Current study	9 healthy volunteers (38.2 ± 15.6)	Dual-tuned sodium and proton-tuned bilateral coil, (Rapid Biomedical) / 3 T (MR750, GE Healthcare)	Large ROI covering as much FGT as possible, small ROI with a fixed size (5 mm in diameter) covering the area with the highest sodium signal	LROI^b^: 52.7 ± 18.6 mmol/L SROI^b^: 71.8 ± 25.5 mmol/L	Not applicable

*FGT* Fibroglandular tissue, *NACT* Neoadjuvant chemotherapy, *NND* Name not disclosed, *NR* Not reported, *pCR* Pathological complete response, *ROI* Region of interest

^a^Mean age of eight volunteers recruited for both the sequence optimisation and three for B_0_ and B_1_ + inhomogeneity and partial volume effect study: 29 ± 5 years

^b^Static magnetic field (B0), transmit radiofrequency field (B_1_ +) and partial volume-effect-corrected values

The TSCs of FGT of the breast have not been reported at 3 T. Notably, our mean TSC values for FGT are slightly higher (LROI, 55.7 ± 16.3 mmol/L (mean ± standard deviation); SROI: 75.4 ± 21.9 mmol/L) in comparison to the previous publications at 1.5 and 7 T that reported mean sodium concentrations ranging between 34 and 49 mmol/L (Table 3). Not all authors described the sampling methods; those who did [6, 8] used larger ROIs. Differences may partly be explained by different coils and ROI sampling methods. Indeed, the use of large ROI is more susceptible to partial volume effects due to the inclusion of adipose tissue which does not contribute towards sodium signal. We are not aware of publications that report FGT FASC values. Our mean FASC values for FGT are slightly higher (LROI, 17.2 ± 6.6 mmol/L (mean ± standard deviation); SROI: 25.2 ± 10.7 mmol/L) than the intracellular sodium concentration in healthy mammalian cells (5−15 mmol/L) [1, 20], but nevertheless lower than the reported mean FASCs in tumours (*e.g.*, ovarian cancer, 20.5 ± 9.9 mmol/L) [1]).

Interestingly, we found that both the FGT TSC and FASC values were significantly different among females with non-dense and dense breasts except for minimum TSC LROI and mean SROI FASC values. The fact that the difference was not seen when minimum LROI measurements were used is likely caused by the inadvertent inclusion of adipose tissue where the sodium values are lower. The TSC measurements inversely correlated with age and but not FGT ADC values. We hypothesise that the inverse correlation likely reflects the age-associated decline in breast density [20]; with age, there is involution of fibroglandular tissue and increase in fatty tissue resulting in reduced TSC values. The inverse correlation between high TSC and low ADC values has been described earlier [6]; the small number (*n* = 12) of evaluable ADC maps in our sample limits the conclusions that can be drawn from our results.

The study is limited by the small sample consisting of only healthy volunteers. Indeed, a larger sample size, preferably with participants from diverse backgrounds and age groups, would have increased the statistical power and generalisability. Further research comparing the effect of different ROI sampling methods and the selected descriptive value in patients with breast lesions is warranted to explore whether our findings can be confirmed in patients. Indeed, research in patients is needed to set standardised criteria for ROI placement for lesion characterisation and treatment response evaluation. Only two readers participated in the reproducibility study. Research in the future in patient should evaluate the effect of reader training and experience on the reproducibility as they may introduce subjectivity and variability. Furthermore, potential advancements is technology, such as improved coils, imaging sequences and denoising techniques, may enhance the clinical applicability of ^23^Na MRI in future. In addition, more research is needed to evaluate whether some individuals with dense breasts might have higher FGT TSC and whether this might be a risk factor for breast cancer. Indeed, sodium contributes to hallmarks of cancer [4, 11, 21, 22], and therefore, higher TSC might be associated with higher breast cancer risk. It would therefore be of interest to evaluate whether the breast cancer risk of some subpopulations could be better appreciated with TSC or FASC values or with a combination of TSC or FASC values and breast density than breast density alone.

To conclude, we found that the chosen sampling method and the selected descriptive parameter affect the measured total sodium and fluid-attenuated sodium concentrations. Our results suggest that females with dense breasts have higher FGT TSC and FASC possibly due to higher cellularity.

### Supplementary Information

**Supplemenatary Material 1. **

## Data Availability

All the relevant data is included in the manuscript. Imaging data is not shareable.

## Abbreviations

ADC: Apparent diffusion coefficient
CI: Confidence interval
DICOM: Digital imaging and communications in medicine
FASC: Fluid-attenuated sodium concentration
FGT: Fibroglandular tissue
LROI: Large region of interest
MRI: Magnetic resonance imaging
ROI: Region of interest
SROI: Small region of interest
TSC: Total sodium concentration
