# Supplementary material for: 23Na MRI: inter-reader reproducibility of normal fibroglandular sodium concentration measurements at 3 T
Source: Eur Radiol Exp. 2024 Jun 10;8:75. doi: 10.1186/s41747-024-00465-x (PMC11162986; doi:10.1186/s41747-024-00465-x)
Supplement: Supplementary file 1 — Supplemenatary Material 1. [file 41747_2024_465_MOESM1_ESM.pdf]

Supplementary Table 1. The effect of phantom stratification. B1-corrected mean, minimum and maximum total sodium concentrations (TSCs) and mean fluid-attenuated sodium concentration (FASC) of healthy volunteers' fibroglandular tissue on a breast level. The results are shown for the readers' large and small regions of interest (ROIs). P-value denotes the statistical difference between the non-B1-corrected and B1-corrected measurements.

		Mean TSC			Minimum TSC			Maximum TSC			Mean FASC		
ROI method	Reader	With phantom stratification (mmol/L)	Without phantom stratification (mmol/L)[a]	*p*-value	With phantom stratification (mmol/L)	Without phantom stratification (mmol/L)[a]	*p*-value	With phantom stratification (mmol/L)	Without phantom stratification (mmol/L)[a]	*p*-value	With phantom stratification (mmol/L)	Without phantom stratification (mmol/L)[a]	*p*-value
Large ROI	Reader 1	55.7 ± 16.3	NA	NA	20.4 ± 6.1	NA	NA	82.5 ± 22.7	NA	NA	17.2 ± 6.6	NA	NA
	Reader 2	60.4 ± 17.2	53.1 ± 16.0	0.014	30.3 ± 8.5	26.7 ± 8.6	0.017	82.4 ± 22.4	72.6 ± 21.4	0.016	18.9 ± 7.3	16.0 ± 6.9	0.01
Small ROI	Reader 1	75.4 ± 21.9	NA	NA	68.4 ± 22.2	NA	NA	81.5 ± 22.1	NA	NA	25.2 ± 10.7	NA	NA
	Reader 2	76.5 ± 20.6	67.5 ± 19.7	0.028	70.9 ± 20.2	62.6 ± 18.7	0.017	81.0 ± 21.3	71.4 ± 20.6	0.016	25.1 ± 10.2	21.4 ± 10.2	0.008

[a] The phantom's signal intensity and B1 measurement measured by Reader 1 was used to minimise the effect of differences caused by phantom ROI placement that contributes to B1-corrected measurements.
NA not applicable.

Supplementary Table 2. Mean, minimum and maximum total sodium concentrations of healthy volunteers' fibroglandular tissue with and without B1 corrections on a breast level. The results are shown for the readers' large and small regions of interest (ROIs). P-value denotes the statistical difference between the non-B1-corrected and B1-corrected measurements.

		Mean			Minimum			Maximum		
ROI method	Reader	Non-B1-corrected (mmol/L)	B1-corrected[a] (mmol/L)	*p*-value	Non-B1-corrected (mmol/L)	B1-corrected[a] (mmol/L)	*p*-value	Non-B1-corrected (mmol/L)	B1-corrected[a] (mmol/L)	*p*-value
Large ROI	Reader 1	96.5 ± 30.5	55.7 ± 16.3	< 0.001	34.4 ± 7.9	20.4 ± 6.1	< 0.001	144.4 ± 46.6	82.5 ± 22.7	< 0.001
	Reader 2	105.4 ± 33.8	60.4 ± 17.2	< 0.001	54.1 ± 19.5	30.3 ± 8.5	< 0.001	143.2 ± 42.3	82.4 ± 22.4	< 0.001
Small ROI	Reader 1	132.2 ± 44.7	75.4 ± 21.9	< 0.001	120.2 ± 45.2	68.4 ± 22.2	< 0.001	142.5 ± 45.3	81.5 ± 22.1	< 0.001
	Reader 2	133.3 ± 40.3	76.5 ± 20.6	< 0.001	123.9 ± 40.3	70.9 ± 20.2	< 0.001	140.9 ± 41.2	81.0 ± 21.3	< 0.001

[a] The phantom's signal intensity and B1 measurement measured by Reader 1 was used to minimise the effect of differences caused by phantom ROI placement that contributes to B1-corrected measurements.
